# Frequency specific alterations of the degree centrality in patients with acute basal ganglia ischemic stroke: a resting-state fMRI study

**DOI:** 10.1007/s11682-023-00806-1

**Published:** 2023-10-12

**Authors:** Hao Chen, Linlin Zhan, Qianqian Li, Chaoguo Meng, Xuemei Quan, Xiaoling Chen, Zeqi Hao, Jing Li, Yanyan Gao, Huayun Li, Xize Jia, Mengting Li, Zhijian Liang

**Affiliations:** 1 Department of Neurology, The First Affiliated Hospital of Guangxi Medical University, Nanning, China; 2 Faculty of Western Languages, Heilongjiang University, Heilongjiang, China; 3 Department of Neurology, The People’s Hospital of Guangxi Zhuang Autonomous Region, Nanning, China; 4 Department of Radiology, The First Affiliated Hospital of Guangxi Medical University, Nanning, China; 5 School of Teacher Education, Zhejiang Normal University, Jinhua, China; 6 Key Laboratory of Intelligent Education Technology and Application of Zhejiang Province, Zhejiang Normal University, Jinhua, China

**Keywords:** Ischemic stroke, Basal ganglia, Resting state fMRI, Degree centrality, Frequency-specific

## Abstract

This study intended to investigate the frequency specific brain oscillation activity in patients with acute basal ganglia ischemic stroke (BGIS) by using the degree centrality (DC) method. A total of 34 acute BGIS patients and 44 healthy controls (HCs) underwent resting-state functional magnetic resonance imaging (rs-fMRI) scanning. The DC values in three frequency bands (conventional band: 0.01–0.08 Hz, slow‑4 band: 0.027–0.073 Hz, slow‑5 band: 0.01–0.027 Hz) were calculated. A two-sample *t*-test was used to explore the between-group differences in the conventional frequency band. A two-way repeated-measures analysis of variance (ANOVA) was used to analyze the DC differences between groups (BGIS patients, HCs) and bands (slow‑4, slow‑5). Moreover, correlations between DC values and clinical indicators were performed. In conventional band, the DC value in the right middle temporal gyrus was decreased in BGIS patients compared with HCs. Significant differences of DC were observed between the two bands mainly in the bilateral cortical brain regions. Compared with the HCs, the BGIS patients showed increased DC in the right superior temporal gyrus and the left precuneus, but decreased mainly in the right inferior temporal gyrus, right inferior occipital gyrus, right precentral, and right supplementary motor area. Furthermore, the decreased DC in the right rolandic operculum in slow-4 band and the right superior temporal gyrus in slow-5 band were found by post hoc two-sample *t*-test of main effect of group. There was no significant correlation between DC values and clinical scales after Bonferroni correction. Our findings showed that the DC changes in BGIS patients were frequency specific. Functional abnormalities in local brain regions may help us to understand the underlying pathogenesis mechanism of brain functional reorganization of BGIS patients.

**Supplementary Information:**

The online version contains supplementary material available at 10.1007/s11682-023-00806-1.

## Introduction

Ischemic stroke (IS) has a high mortality and disability rate (Zhou et al., 2019), and its survivors often suffer from motor, cognitive and/or other neurological dysfunctions (Yao et al., 2020; Zhao et al., 2018a). Previous neuroimaging studies showed that functional impairments or compensations of brain regions and functional networks in IS, also known as functional reorganization of brain, are correlated with the neurological dysfunctions (Barrett et al., 2019; Favre et al., 2014; Li et al., 2020a). In addition, lesion inference analysis demonstrated that the functional impairments after IS were closely related to the lesion locations (Zavaglia et al., 2015). The basal ganglia region is a common location of stroke and the basal ganglia ischemic stroke (BGIS) has long-term impacts on patients’ motor and cognitive functions (Li et al., 2020b). However, BGIS has not received much research attention and the neuroimaging features and potential neurobiological mechanism of brain functional reorganization in BGIS remain unclear.

Resting-state functional magnetic resonance imaging (rs-fMRI) measures spontaneous low-frequency oscillations (LFO) of the blood oxygen level dependent (BOLD) signal and has been widely employed to investigate the changes of brain function in patients and healthy people (Biswal et al., 1995; Guan et al., 2019; Mohanty et al., 2019; Sang et al., 2012). Rs-fMRI is a noninvasive and task-free technique, and is suitable for the study on functional reorganization after IS, having the advantage of revealing the mechanism of the abnormalities in brain regions and functional networks caused by stroke (Zhao et al., 2018b). Degree centrality (DC) is an analytical method to characterize the local properties of the rs-fMRI signals by measuring the level of a given brain voxel (node) connected directly by the other voxels (Buckner et al., 2009; Zuo et al., 2012). It describes the importance of local brain regions in the whole brain network. At present, the DC method has been widely used in exploring the brain functional reorganization in various neurological diseases (Li et al., 2018, 2021). In the stroke domain, scholars have investigated the functional reorganization in chronic ischemic stroke patients with different lesion locations, including cortex and pons, but rare in basal ganglia region (Jiang et al., 2019; Mazrooyisebdani et al., 2018; Shi et al., 2017). Recently, a rs-fMRI study (Yao et al., 2020) on patients with basal ganglia damage (including ischemia and hemorrhage) found that, compared with healthy controls (HCs), patients had abnormalities of functional networks in multiple brain regions, including angular gyrus, right supramarginal gyrus, and the DC value decreased in the right supramarginal gyrus was correlated with cognitive dysfunction.

However, the above DC studies of IS patients mainly explore LFO activity in the conventional band (0.01–0.08 Hz) (Jiang et al., 2019; Shi et al., 2017; Yao et al., 2020). Recent rs-fMRI studies dedicated to explore LFO in slow-5 band (0.01-0.027Hz), slow-4 band (0.027-0.073Hz), slow-3 band (0.073-0.198Hz) and slow-2 band (0.198-0.25Hz) (Zuo et al., 2010), and found that the oscillations in slow-5 and slow-4 bands primarily correlated with the neural activity of gray matter signals. It is suggested that oscillations in these two frequency bands are the most useful ones in exploring relationship between functional processing disorders (Guan et al., 2019; Zuo et al., 2010). The frequency specific characteristics have been observed in functional connectivity studies, which showed different patterns in different frequency bands. Previous research (Zhu et al., 2015) found that the LFO detected by the slow-5 band was more extensive in patients with IS, while another study showed that the slow-4 band may be more sensitive in detecting functional connectivity alterations in patients with subclinical language deficit after stroke (Mohanty et al., 2019). Many studies have revealed the frequency specific brain neural activity of neurological diseases (Gu et al., 2019; Liao et al., 2021; Mohanty et al., 2019; Yu et al., 2014; Zhu et al., 2015). However, there are no study that have explored the characteristics of brain neural activity in patients with acute BGIS using the DC method in multiple frequency bands.

We hypothesized that changes of DC values in patients with acute BGIS were frequency specific. Therefore, this study used the DC method to investigate the characteristics of node properties in brain networks of patients with acute BGIS in conventional, slow-4 and slow-5 bands. Moreover, the potential neurobiological mechanism of brain functional reorganization in BGIS was also explored by correlation analyses between the abnormal DC values and clinical indicators of patients. We hope that the findings in present study could deepen our understanding of the underlying pathogenesis mechanism of BGIS and thus provide relatively targeted guidance for the treatments of BGIS patients.

## Materials and methods

### Participants

From May 2019 to December 2020, 43 patients with acute BGIS from the First Affiliated Hospital of Guangxi Medical University were continuously enrolled in this study. At the same time, 47 HCs were recruited from the community. This study was approved by the Ethics Committee of the First Affiliated Hospital of Guangxi Medical University. Written informed consent was obtained from all participants.

The inclusion criteria for patients were as follows: (1) age between 30 to 75 years; (2) right-handed; (3) first-ever stroke and single lesion was located in the basal ganglia region that was defined to include basal ganglia, capsula interna and corona radiata; (4) MRI was performed within 10 days after stroke; (5) no other serious diseases; (6) no history of other neuropsychiatric diseases; (7) no history of drug or alcohol abuse. The inclusion criteria for HCs were as follows: (1) age-matched with the BGIS patients; (2) right-handed; (3) no history of neuropsychiatric diseases, such as stroke, epilepsy, depression and so on.

The exclusion criteria for all participants were as follows: (1) contraindicated of MRI examinations, such as claustrophobia and metal implants; (2) MRI images showed that participants had abnormal signals caused by epilepsy, cerebral hemorrhage or other neurological disorders; (3) MRI data loss; (4) excessive head movement during the examination and/or poor data quality (see below for details). Specific exclusion criteria for the patients with BGIS were: (1) unable to complete neurological function assessment. For example, patients with Broca aphasia or Wernicke aphasia, auditory or visual disorders would be excluded; (2) as revealed by previous studies(Erdoğan et al., 2016; Tong et al., 2019), perfusion abnormality could influence rs-fMRI signals and should be corrected. Therefore, patients with significant perfusion changes would be excluded.

Nine BGIS patients (data loss: 1, head movement: 2, MRI image damaged: 1, incomplete scanning of cerebellum: 5) and three HCs (data loss: 1, head movement: 1, incomplete scanning of cerebellum: 1) were excluded according to the above criteria, leaving 34 patients and 44 HCs in the final analysis.

### Demographic and clinical data

All patients’ demographic information, including age, gender, education, disease history, were collected. The information of lesion location in patients was obtained based on MRI data. The National Institutes of Health Stroke Scale (NIHSS) was used to quantify the stroke severity and neurological deficits (Kasner 2006). Meanwhile, the motor function was assessed by the Fugl-Meyer Assessment (FMA) (Fugl-Meyer et al., 1975) and the activities of daily living were evaluated with the Barthel Index (BI) (Leung et al., 2007).

### MRI data acquisition

MRI data was collected for each subject using a Siemens Prisma 3.0 T MR scanner (Siemens, Erlangen, Germany) at the First Affiliated Hospital of Guangxi Medical University. During the data acquisition, participants were required to be awake with eyes closed in a supine position, and head movement of participants was restricted using foam padding.

Resting-state images were acquired using echoplanar imaging (EPI) sequence with the following parameters: repetition time (TR) = 2,000 ms, echo time (TE) = 35 ms, slices number = 40, thickness/gap = 3/0 mm, flip angle (FA) = 90°, field of view (FOV) = 240 × 240 mm^2^, voxel size = 2.6 × 2.6 × 3mm^3^, matrix = 64 × 64. This session lasted for 6 minutes and 12 seconds. The anatomical 3D-MPRAGE T1-weighted images (T1WI) were recorded by magnetization prepared rapid gradient echo: TR = 2300 ms, TE = 2.98 ms, inversion time = 900 ms, slices number = 176, thickness/gap = 1/0 mm, FOV = 256 × 256 mm^2^, voxel size = 1 × 1× 1 mm^3^, matrix = 256 × 256. This session lasted for 5 minutes and 21 seconds.

### MRI data preprocessing

All algorithms are implemented in Matlab R2018a (https://uk.mathworks.com/products/matlab). Rs-fMRI data preprocessing and statistical analyses were using the Statistical Parametric Mapping (SPM, version 12) software (http://www.fil.ion.ucl.ac.uk/spm) and Resting-State fMRI Data Analysis Toolkit plus (RESTplus) V1.25 (http://restfmri.net/forum/restplus) software (Jia et al., 2019). Prior to preprocessing, we conducted systematic quality inspections of each participant’s resting-state data. One patient was excluded from further analysis because of the damaged image. The main steps of preprocessing included: (1) the first 10 time points of each subject were removed to allow the subject to adapt to the scanning noise and avoid the non-equilibrium effects of magnetization; (2) slice timing correction for adjusting acquisition delay between slices; (3) realignment was conducted in which all the volumes were aligned to the first volume. Participants with excessive head motion (exceeding 3mm or 3^°^) were excluded from further analysis (two patients and one HC were excluded); (4) the spatial normalization was conducted. Namely, the structural image was coregistered to the mean functional image after the head motion correction; the transformed structural image was then segmented into gray matter, white matter, cerebrospinal fluid, bone, soft tissue and air by using a new segmentation algorithm. Then the functional images after head motion correction were normalized to the MNI space using the normalization parameters generated during the new segmentation (Mechelli et al., 2005); (5) removing the linear trend of the time series; (6) regressing out nuisance variables, including cerebrospinal fluid signals, white matter signals and the Friston-24 head motion parameters (Friston et al., 1996); (7) a band pass filter was used to extract signals in the conventional frequency band (0.01–0.08 Hz), slow-4 band (0.027–0.073 Hz) and slow-5 band (0.01–0.027 Hz).

### DC calculation

DC analysis was performed by using the RESTplus V1.25 software. The DC was defined by calculating the correlation between each voxel and every other voxel in the whole brain (Buckner et al., 2009). Pearson’s correlation coefficients (*r*) were computed to measure the time courses between a voxel and all voxels. The DC values at the voxel level of the whole brain were first calculated with the correlation threshold set at *r* > 0.25 (Shi et al., 2017; Yao et al., 2020). Then the weighted DC strength of a voxel was computed as the sum of the connections between a given brain voxel and all other voxels. Finally, all standardization DC maps, in which DC values were divided by the whole-brain average DC value, were spatial smoothed with a Gaussian kernel of 4 mm full-width at half-maximum (FWHM). To investigate the changes of brain oscillation activity in acute BGIS patients, the DC values of the conventional band, slow-4 band and slow-5 band were calculated.

For easy comparison, the lesion on the right was flipped from right to left after the DC calculation (Park et al., 2011). The flips were performed on DC maps.

### Statistical analysis

SPSS software version 23.0 (IBM, Armonk, NY, USA) was used for statistical analyses. Categorical variables are presented as n (%), and the chi-square test was used to test the difference between patients and HCs. The continuous variables are presented as the mean ± standard deviation (SD) or median and interquartile range (IQR), and the independent-sample *t*-test was performed to test the difference between patients and HCs. All tests of demographic information were two-tailed and *p* < 0.05 was considered significant.

First, a two-sample *t*-test was performed to examine differences between the two groups in the conventional band using RESTplus V1.25. Then, to examine the effects of group and frequency band in DC, a two-way repeated-measures analysis of variance (ANOVA) was conducted by using SPM12. Group (BGIS patients and HCs) served as a between-subject factor, and frequency band (slow-4 band and slow-5 band) as a repeated-measures factor. The two-way repeated measures ANOVA was also used to analyze the interaction effects between the group and frequency band. Gender and mean frame-wise displacement (FD) (Jenkinson et al., 2002) were used as covariates. The significance threshold corrections of all the statistical maps (*T*-amps and *F*-maps) were based on the False Discovery Rate (FDR) with *p* value < 0.05, cluster size > 50 by Data Processing & Analysis for Brain Imaging (DPABI) V5.1 (http://rfmri.org/dpabi). Finally, the post hoc two-sample *t*-test was conducted in the regions that exhibit significant main effects and interaction between group and frequency band (*p* < 0.05, FDR correction).

The DC values in brain regions of BGIS and HCs of group main effect results and post hoc results were extracted. Spearman or Pearson correlation analysis was used to analyze the relationship between the DC values and clinical indicators of patients (including the NIHSS, FMA and BI scores) to identify the relationships between the DC values and clinical indicators. The statistical significance threshold was set at *p* < 0.05. Bonferroni correction was used to correct the correlation results.

## Results

### Demographic and clinical characteristics

There were 34 patients and 44 HCs enrolled in the final analysis. The demographic and clinical information of all participants were summarized in Table 1. No significant differences were observed in age (*p* = 0.736) and education (*p* = 0.053) between BGIS patients and HCs. However, gender (*p* = 0.007), hypertension (*p* < 0.001) and diabetes (*p* = 0.041) were observed between BGIS and HCs groups. The maps of stroke lesion were shown in supplementary materials (See Fig. S1 in the supplementary materials). All patients completed the NIHSS, FMA and BI scores. Overall, BGIS patients reported a median NIHSS score of 3 (interquartile range, 2–6), a mean FMA score of 74.910 ± 18.907, and a mean BI score of 75.441 ± 20.389 in this study.

**Table 1:** Demographic and clinical characteristics of the subjects

	BGIS (*n* = 34)	HCs (*n* = 44)	*t*/χ^2^	*P* value
Age (years)	56.500 ± 10.999^**a**^	55.340 ± 11.485^**a**^	0.450	0.736^**c**^
Gender(male/female)	25/9	19/25	7.184	0.007^**d**^
Education (years)	11.500 ± 3.587^**a**^	12.140 ± 3.008^**a**^	-0.852	0.053^**c**^
Hypertension(n./%)	26 (76.471%)	7 (15.909%)	28.820	< 0.001^**d**^
Diabetes(n./%)	9 (26.471%)	4 (9.091%)	4.171	0.041^**d**^
Lesion side (right/left)	14/20	-		-
Lesion location				
Basal ganglia(n./%.)	1 (2.941%)	-		-
Basal ganglia-capsula interna(n./%)	4 (11.765%)	-		-
Basal ganglia-corona radiata(n./%)	20 (58.824%)	-		-
Corona radiata(n./%)	9 (26.471%)	-		-
NIHSS score	3 (2–6)^**b**^	-		-
FMA score	74.910 ± 18.907^**a**^	-		-
BI score	75.441 ± 20.389^**a**^	-		-

a, mean ± standard deviation; b, median (interquartile range); c, The *P* value was obtained by the independent-sample *t*-test; d, The *P* value was obtained by the Chi-Square test

Abbreviations: BGIS, basal ganglia ischemic stroke; HCs, healthy controls; NIHSS, National Institutes of Health Stroke Scale; FMA, Fugl-Meyer Assessment; BI, Barthel Index

### DC analysis in conventional frequency band

**Fig. 1:**
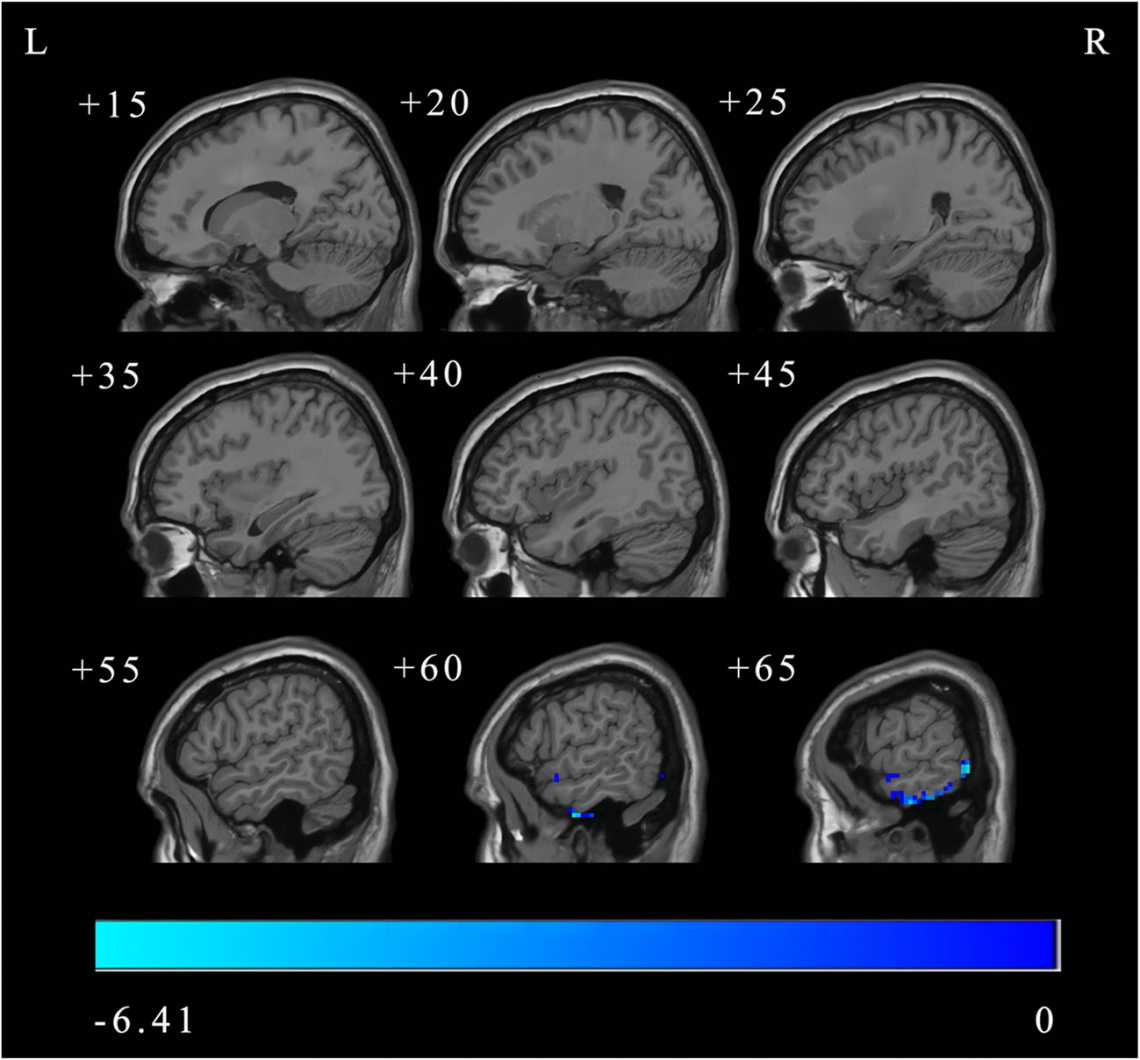
DC difference in conventional frequency band between BGIS and HCs groups (FDR correction, *p* < 0.05, cluster size > 50). L, left; R, right; DC, degree centrality; BGIS, basal ganglia ischemic stroke; HCs, healthy controls; FDR, False Discovery Rate

The result showed that the DC value in the right middle temporal gyrus was decreased in BGIS patients compared with HCs in conventional frequency band (FDR correction, *p* < 0.05, cluster size > 50) (Fig. 1 and Table 2).

**Table 2:** DC difference in conventional frequency band between BGIS and HCs groups (FDR correction, *p* < 0.05, cluster size > 50)

Brain regions (AAL)	Number of voxels	MNI coordinate (x, y, z)	Peak *T* value
Right middle temporal gyrus	263	(60, -6, 39)	-6.4101

Abbreviations: DC, degree centrality; BGIS, basal ganglia ischemic stroke; HCs, healthy controls; FDR, False Discovery Rate; AAL, Anatomical Automatic Labeling; MNI, Montreal Neurological Institute

### Main effect of the frequency band and group

**Fig. 2:**
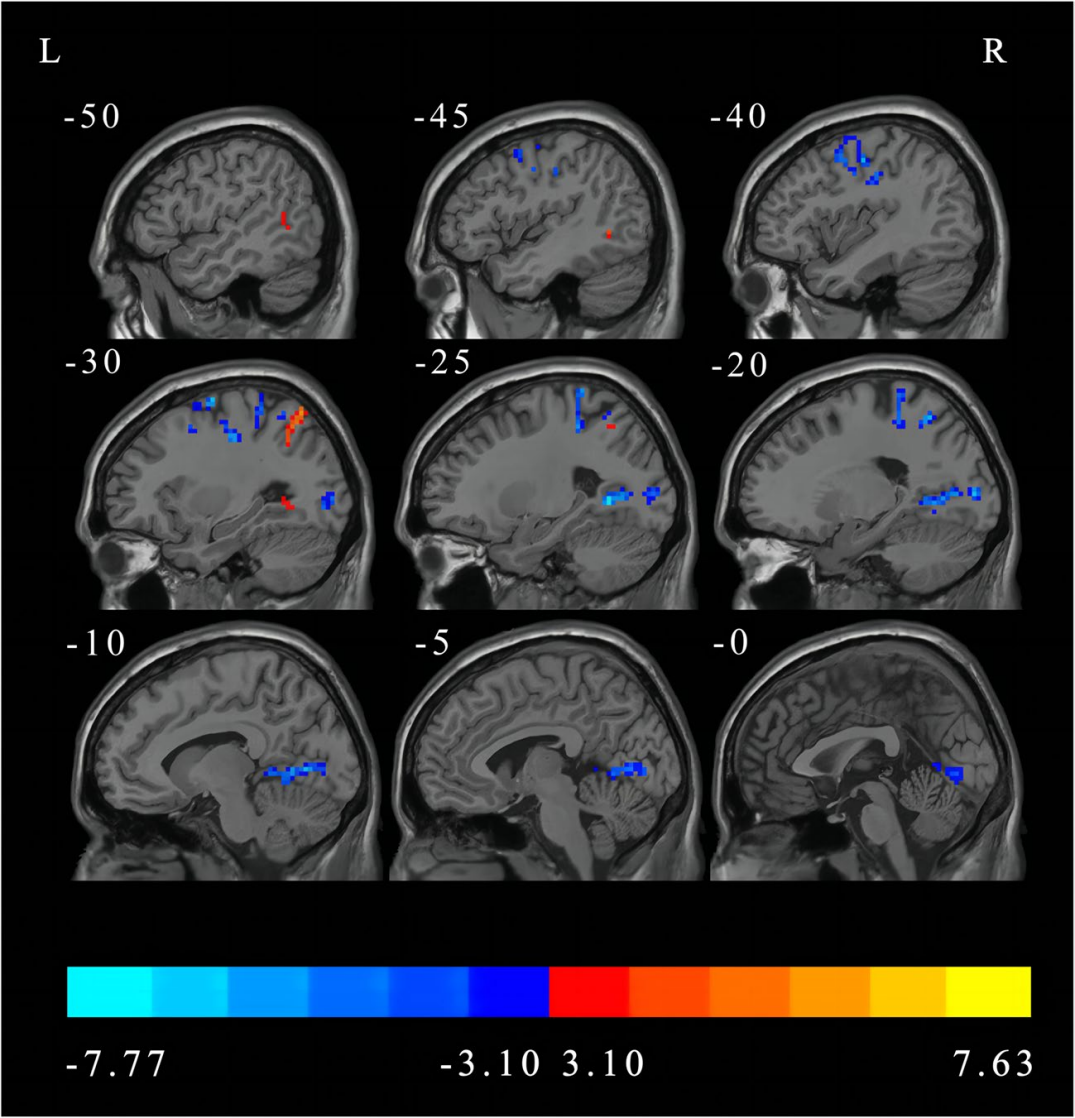
Main effect of the frequency band factor on DC (FDR correction, *p* < 0.05, cluster size > 50). L, left; R, right; DC, degree centrality; FDR, False Discovery Rate

The main effect of the frequency band is shown in Fig. 2 and Table 3. Compared with the slow-5 band, the slow-4 band showed significantly decreased DC in the right fusiform, left precuneus, right middle occipital gyrus, bilateral precentral gyrus, left superior parietal gyrus, and left postcentral gyrus, but significantly increased DC in the bilateral middle temporal gyrus, right insula, and right supramarginal gyrus (FDR correction, *p* < 0.05, cluster size > 50).

**Table 3:** The main effect of frequency band on DC revealed by two-way repeated-measures ANOVA (FDR correction, *p* < 0.05, cluster size > 50)

Brain regions (AAL)	Number of voxels	MNI coordinate (x, y, z)	*T* value
Slow-4 < Slow-5			
Right fusiform	265	(51, -81, -9)	-7.0484
Left precuneus	340	(-24, -57, 3)	-7.0578
Right middle occipital gyrus	64	(39, -78, 12)	-5.5733
Right middle occipital gyrus	58	(27, -75, 27)	-6.2694
Right precentral gyrus	127	(39, -15, 33)	-7.3764
Left precentral gyrus	166	(-33, -18, 45)	-7.4015
Left superior parietal gyrus	71	(-21, -57, 57)	-6.5393
Left postcentral gyrus	86	(-18, -36, 60)	-7.7746
Slow-4 > Slow-5			
Left middle temporal gyrus	53	(-63, -57, 0)	7.6261
Right middle temporal gyrus	106	(54, -54, 9)	5.2762
Right insula	57	(33, -21, 21)	7.0722
Right supramarginal gyrus	102	(51, -33, 24)	5.9944

Abbreviations: DC, degree centrality; ANOVA, analysis of variance; FDR, False Discovery Rate; AAL, Anatomical Automatic Labeling; MNI, Montreal Neurological Institute

**Fig. 3:**
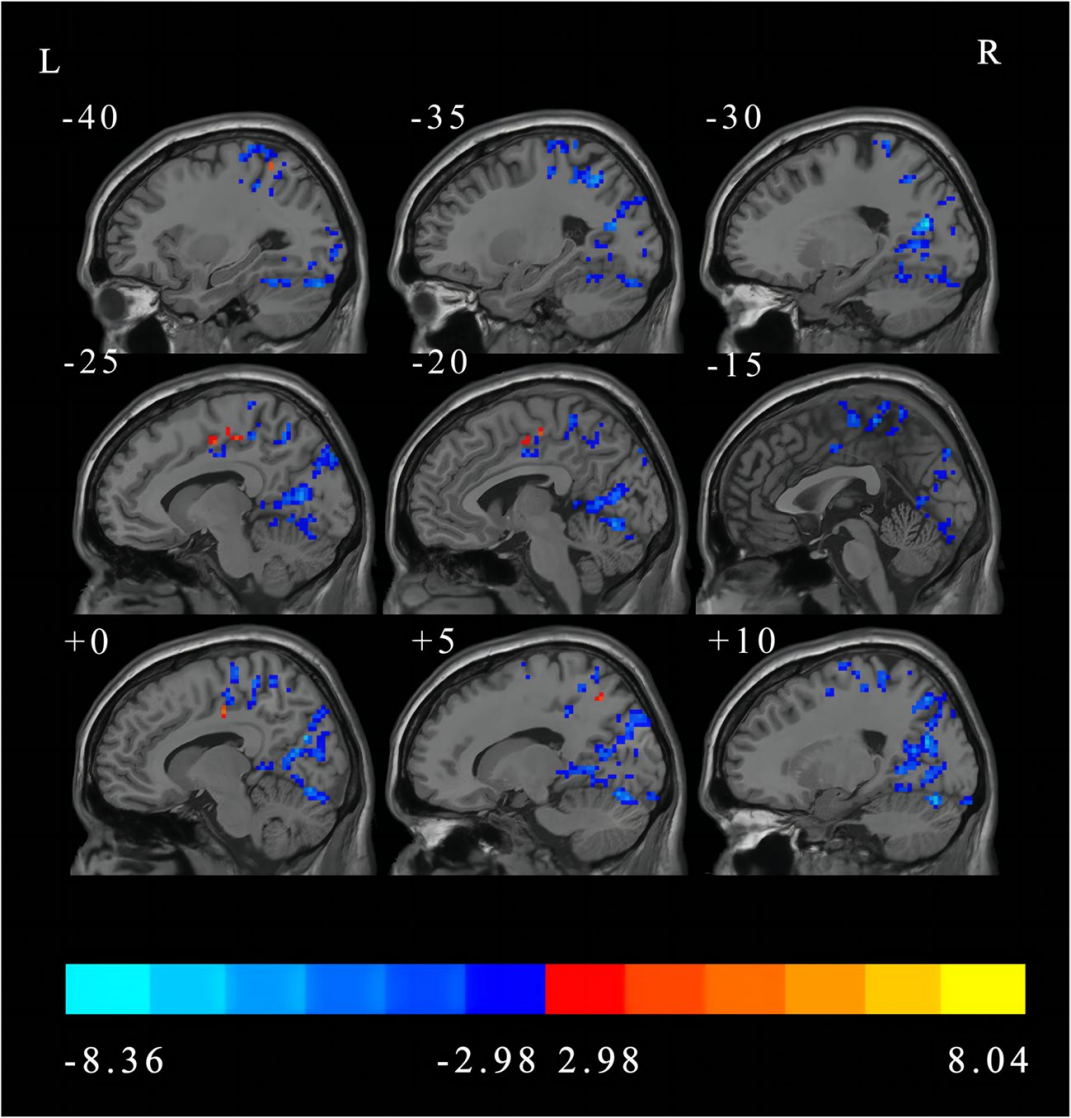
Main effect of the group factor on DC (FDR correction, *p* < 0.05, cluster size > 50). L, left; R, right; DC, degree centrality; FDR, False Discovery Rate

The main effect of the group is shown in Fig. 3 and Table 4. Compared with the HCs, the BGIS patients exhibited significantly increased DC in the right superior temporal gyrus and the left precuneus, but decreased DC in the right calcarine fissure, right inferior temporal gyrus, right inferior occipital gyrus, right precentral, left postcentral gyrus, right supplementary motor area, and the left paracentral lobule (FDR correction, *p* < 0.05, cluster size > 50).

**Table 4:** The main effect of group on DC revealed by two-way repeated-measures ANOVA (FDR correction, *p* < 0.05, cluster size > 50)

Brain regions (AAL)	Number of voxels	MNI coordinate (x, y, z)	*T* value
BGIS > HCs			
Right superior temporal gyrus	83	(69, -6, 6)	6.7662
Left precuneus	206	(-15, -51, 54)	8.0472
BGIS < HCs			
Right calcarine fissure	1749	(-21, -69, 18)	-8.3657
Right inferior temporal gyrus	80	(54, -69, -9)	-6.4251
Right inferior occipital gyrus	61	(33, -99, 6)	-7.1392
Right precentral	731	(45, -15, 54)	-8.0385
Left postcentral gyrus	69	(-48, -15, 51)	-5.5047
Left postcentral gyrus	140	(-36, -36, 45)	-6.5041
Right supplementary motor area	134	(6, -21, 69)	-5.9049
Left paracentral lobule	111	(-12, -33, 75)	-6.4616

Abbreviations: DC, degree centrality; ANOVA, analysis of variance; FDR, False Discovery Rate; AAL, Anatomical Automatic Labeling; MNI, Montreal Neurological Institute; BGIS, basal ganglia ischemic stroke; HCs, healthy controls

### Interaction effects and the post hoc two-sample *t*-test

**Fig. 4:**
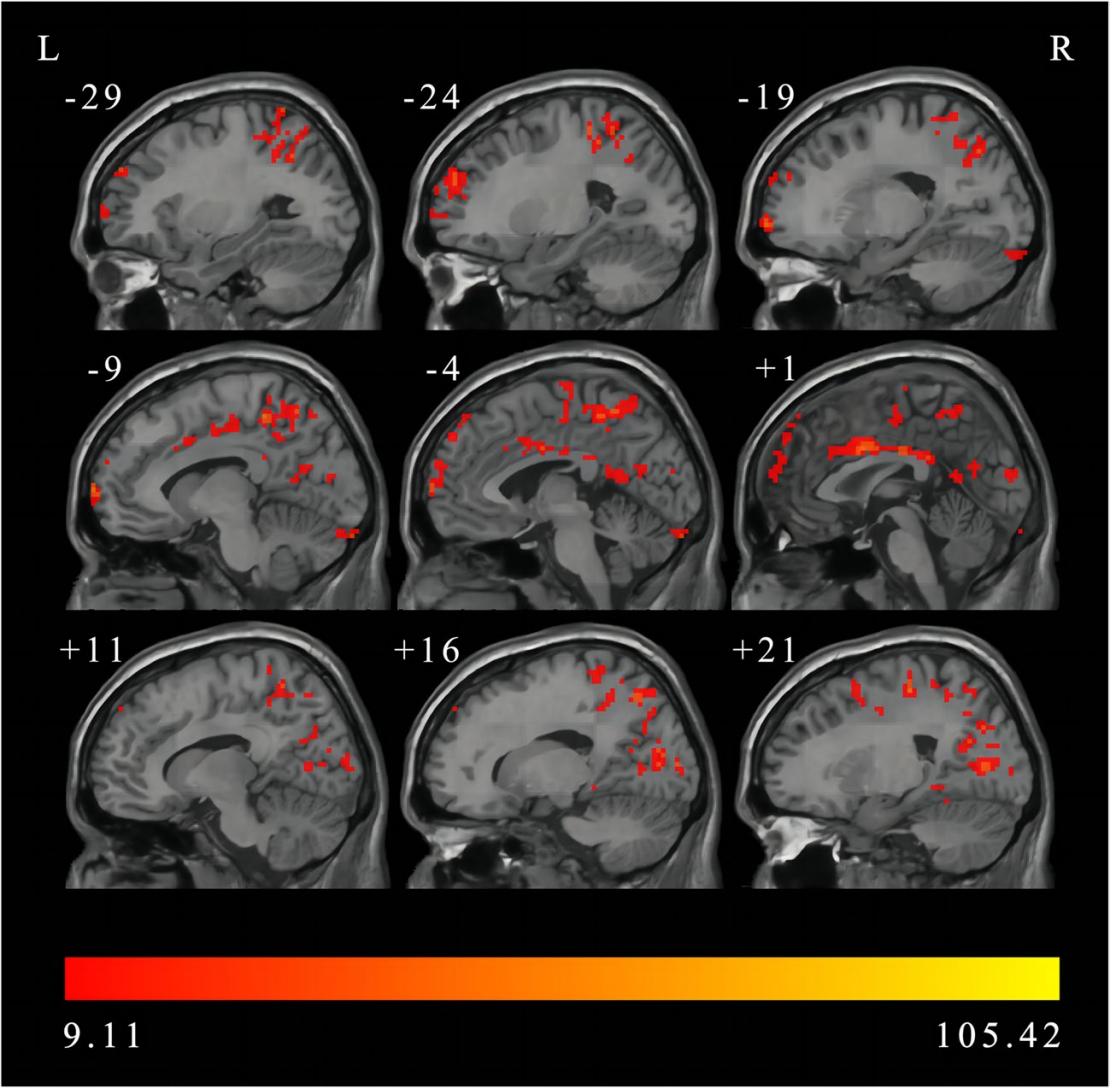
The interaction between frequency bands and group revealed by two-way repeated-measures ANOVA (FDR correction, *p* < 0.05, cluster size > 50). L, left; R, right; DC, degree centrality; ANOVA, analysis of variance; FDR, False Discovery Rate

Significant interaction effects in brain regions in bilateral cerebral hemisphere were observed between the group and frequency band (FDR correction, *p* < 0.05, cluster size > 50). As showed in Fig. 4 and Table 5, left inferior temporal gyrus, left cerebellum crus1, right fusiform gyrus, left superior temporal gyrus, right inferior occipital gyrus, right calcarine fissure and surrounding cortex, right precuneus, left superior frontal gyrus, bilateral median cingulate and paracingulate gyri, right precentral gyrus, bilateral inferior parietal, left superior parietal gyrus, right median cingulate and paracingulate gyri, right superior parietal gyrus were significant interactions between different groups and frequency bands and showed significant interaction effects.

**Table 5:** The interaction between frequency bands and groups on DC by two-way repeated-measures ANOVA (FDR correction, *p* < 0.05, cluster size > 50)

Brain regions (AAL)	Number of voxels	MNI coordinate (x, y, z)	Peak *F* value
Left inferior temporal gyrus	61	(-57, -57, -27)	53.3992
Left Cerebellum Crus1	54	(-6, -96, -24)	49.4503
Right fusiform gyrus	86	(42, -51, 15)	38.5934
Left superior temporal gyrus	79	(-54, -12, -6)	39.3854
Right inferior occipital gyrus	275	(42, -90, -3)	71.0404
Right calcarine fissure and surrounding cortex	51	(15, -90, 9)	25.3759
Right calcarine fissure and surrounding cortex	136	(27, -66, 6)	105.4208
Right precuneus	208	(36, -63, 15)	40.1362
Left superior frontal gyrus, dorsolateral	247	(-15, 69, 0)	54.4945
Left median cingulate and paracingulate gyri	443	(-6, -39, 51)	53.403
Right precentral gyrus	54	(42, 3, 36)	23.0378
Right median cingulate and paracingulate gyri	192	(3, 6, 33)	56.7939
Right inferior parietal, but supramarginal and angular gyri	267	(39, -39, 42)	40.0164
Left superior parietal gyrus	52	(-15, -69, 48)	57.8348
Right median cingulate and paracingulate gyri	79	(6, -15, 48)	29.2486
Right precentral gyrus	120	(33, -3, 48)	41.7986
Right superior parietal gyrus	85	(15, -66, 54)	31.6564
Right precentral gyrus	71	(21, -24, 57)	47.8886

Abbreviations: DC, degree centrality; ANOVA, analysis of variance; FDR, False Discovery Rate; AAL, Anatomical Automatic Labeling; MNI, Montreal Neurological Institute

**Fig. 5:**
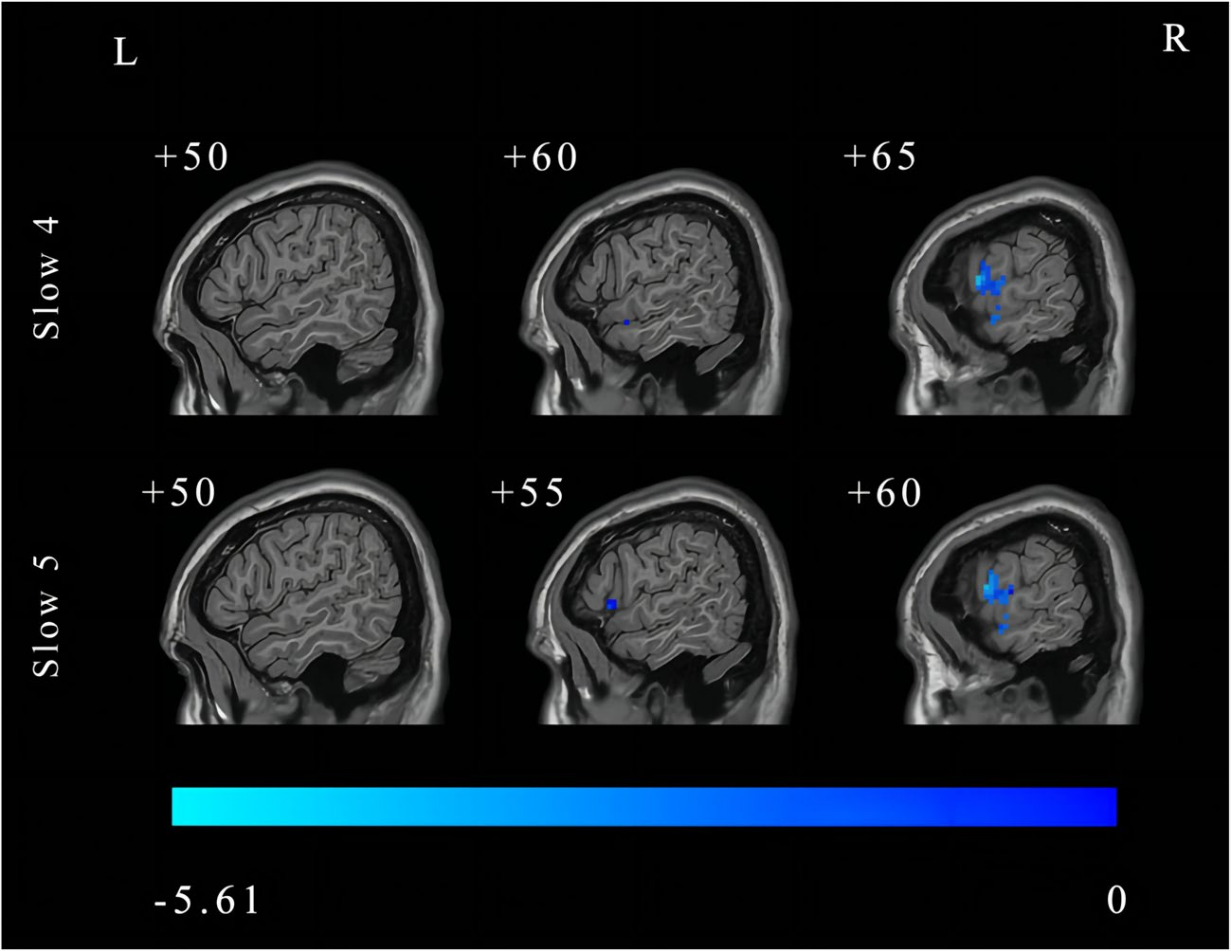
The post hoc two-sample *t*-test in the main effects and interaction between group and frequency band. (FDR correction, *p* < 0.05). L, left; R, right; FDR, False Discovery Rate

Further, a post-hoc *t* test revealed that DC significantly decreased in the right rolandic operculum in the slow-4 band and decreased in the right superior temporal gyrus in the slow-5 band in BGIS patients (FDR correction, *p* < 0.05, cluster size > 50) (Fig. 5 and Table 6).

**Table 6:** The post hoc two-sample *t*-test in the main effect of group (FDR correction, *p* < 0.05)

Brain regions (AAL)	Number of voxels	MNI coordinate (x, y, z)	Peak *T* value
BGIS < HCs			
Slow-4 band			
Right rolandic operculum	58	(66, 6, 12)	-5.4633
Slow-5 band			
Right superior temporal gyrus	62	(69, -6, 6)	-5.6196

Abbreviations: ANOVA, analysis of variance; FDR, False Discovery Rate; AAL, Anatomical Automatic Labeling; MNI, Montreal Neurological Institute; BGIS, basal ganglia ischemic stroke; HCs, healthy controls

All the original *T*-map and *F*-maps were shared online (http://restfmri.net/BGIS_DC.zip).

### Correlation analysis

The DC values in brain regions of BGIS and HCs of group main effect results and post hoc results were extracted and then correlated with the clinical indicators.

In the slow-4 band, the decreased DC value in the right inferior temporal gyrus of BGIS patients negatively correlated with NIHSS scores (*r* = -0.385, *p* = 0.024, uncorrected) while positively correlated with FMA score (*r* = 0.483, *p* = 0.004, uncorrected) and BI score (*r* = 0.361, *p* = 0.036, uncorrected) (Table 7). Nevertheless, the correlation between the clinical indicators and DC value in the slow-4 band in the right inferior temporal gyrus of BGIS stroke patients was no longer significant after Bonferroni correction.

**Table 7:** Correlation between the DC values and clinical indicators in BGIS patients (*p* < 0.05, uncorrected)

Frequency band	Brain regions (AAL)	Clinical indicator	Correlation coefficient
Slow-4 band	Right inferior temporal gyrus	NIHSS	*r* = -0.385, *p* = 0.024
	Right inferior temporal gyrus	FMA	*r* = 0.483, *p* = 0.004
	Right inferior temporal gyrus	BI	*r* = 0.361, *p* = 0.036

Abbreviations: DC, degree centrality; BGIS, basal ganglia ischemic stroke; AAL, Anatomical Automatic Labeling; NIHSS, National Institutes of Health Stroke Scale; FMA, Fugl-Meyer Assessment; BI, Barthel Index

In the slow-5 band, with or without Bonferroni correction, no correlation was found between the DC values of the abnormal brain regions and the NIHSS, FMA and BI scores.

## Discussion

In this study, we explored the abnormalities of LFO activity in acute BGIS patients in multiple frequency bands (the conventional band, slow-4 band and slow-5 band) by using the DC method and analyzed the relationship between DC values and clinical indicators of patients. Compared with HCs, we found that the DC value decreased in the right middle temporal gyrus in the conventional band among BGIS patients. Meanwhile, several brain regions exhibited significant differences between two bands (slow-4 and slow-5 bands) and between two groups. Significant frequency band and group interaction effects were also observed in bilateral brain regions. Additionally, the decreased DC value in the right inferior temporal gyrus in slow-4 band was associated with the NIHSS, FMA, and BI scores (uncorrected). These findings supported our hypothesis that changes of DC values in patients with acute BGIS were frequency specific. We next discuss the theoretical and clinical implications of these findings.

### Difference in DC in conventional frequency band

In the present study, BGIS patients showed significantly decreased DC value in the right middle temporal gyrus in conventional band compared with HCs. The middle temporal gyrus plays an essential role in language processing, emotion management and memory function (Bonilha et al., 2017; Yun et al., 2017). Hillis and his colleagues (Hillis et al., 2006) found that the reperfusion of this region during the 3–5 days post-stroke was associated with improvement in picture naming. A previous study (van Hees et al., 2014) also found that pre-treatment amplitude of low-frequency fluctuation (ALFF) value in the right middle temporal gyrus correlated with greater outcomes of the phonological treatment. Tuo (Tuo et al., 2021) used graph-theory method and found the degree centrality, nodal efficiency, and betweenness centrality of right middle temporal gyrus were significantly lower in asymptomatic patients with carotid plaque than that in HCs, suggesting functional reorganization in response to early brain damage. Additionally, the structural and functional connectivity of the middle temporal gyrus has been found to be involved in a large network (Hsu 2016). The changes of attributes in local regions may correspond to changes in relevant brain functions. Thus, based on prior studies that presented the connection between right middle temporal gyrus and language processing we mentioned above, the decreased DC in the right middle temporal gyrus in the current study may also be one of the reasons which lead to language and cognitive impairments in patients with BGIS.

### Main effect of the frequency bands and group and interaction effects

The main effect of frequency bands revealed that slow-5 band exhibited higher DC values in widespread cortical regions, including the right fusiform, left precuneus, right middle occipital gyrus, bilateral precentral gyrus, left superior parietal gyrus and left postcentral gyrus. The LFOs in specific frequency bands are generated from different neurophysiological mechanisms (Biswal et al., 1995; Zuo et al., 2010). Previous rs-fMRI studies have suggested that the slow-5 band exhibited more extensive LFOs than slow-4 band in the cortical regions (Gu et al., 2019; Yu et al., 2014). Our results are in line with previous studies, showing that the slow-5 band is more likely to detect functional activity in brain regions of the frontal, occipital, and parietal cortices.

The main effect of groups showed that the DC values increased in the right superior temporal gyrus and left precuneus in acute BGIS patients compared with HCs. Increased DC values in insula, caudate and other brain regions have been found in previous rs-fMRI studies on IS, indicating the compensation of functional deficits after brain injury (Jiang et al., 2019; Shi et al., 2017). The heterogeneous clinical characteristics, such as lesion site, stroke severity, and illness duration may lead to different functional compensation (Zavaglia et al., 2015). Moreover, the pathophysiological changes of IS are not very comprehensive in acute stage (Khoshnam et al., 2017). The functional reorganization in different temporal dynamics after IS has been observed in previous studies (Hu et al., 2018; Li et al., 2020b). A study on stroke has demonstrated that new connectivity would reestablish in both peri-infarct areas and other distant regions to compensate for the loss of function in the damaged regions during the processing of functional reorganization after stroke (Clark et al., 2019). The increased DC in our study may be an early compensatory expression of impaired brain function after stroke and can help us understand the possible pathogenesis mechanisms of rehabilitation after dysfunction.

Furthermore, compared with HCs, the decreased DC values were found in the right calcarine fissure, right inferior temporal gyrus, right inferior occipital gyrus, right precentral, left postcentral gyrus, right supplementary motor area, and the left paracentral lobule in acute BGIS patients. The results of this study were similar to previous studies, in which decreased functional connectivity between the precentral gyrus (Chen et al., 2021), occipital cortex (Li et al., 2020b), calcarine (Jiang et al., 2019), paracentral lobule (Zhang et al., 2016) and other regions were also observed in ischemic stroke patients. The supplementary motor area is involved in motor supplementary function (Cona & Semenza, 2017) and the precentral gyrus accounts for motor information processing and plays a key role in executive and body motor functions (Grefkes et al., 2008). An fMRI study using the voxel-mirrored homotopic connectivity (VMHC) method found lower connectivity of the precentral gyrus in 30 left subcortical chronic stroke patients with pure motor deficits than HCs (Tang et al., 2016). Hu and colleagues (Hu et al., 2018) found that compared with subacute stage, the temporal variability of functional connectivity in the precentral gyrus was decreased in acute stage in IS patients with hand motor deficit, and the long-term motor recovery is related to changes in temporal variability of the precentral gyrus. The postcentral gyrus is located in between the central sulcus and postcentral sulcus and is responsible for proprioception processing (Cao et al., 2018). Researchers (Yao et al., 2020) found that the DC value of the postcentral gyrus decreased in patients with chronic BGIS. The cerebral infarction localized to the postcentral gyrus can also cause severe impairment of motor control (Kato & Izumiyama, 2015). The anatomical and physiological mechanisms showed that BGIS can lead to motor and sensory dysfunctions. Therefore, we speculated that the DC values decreased in the precentral gyrus and supplementary motor area and postcentral gyrus in our study may aggravate motor and sensory dysfunctions in BGIS patients.

The interaction effects between the group and frequency band were observed in extensive cortical brain regions of bilateral cerebral hemisphere. In addition to motor dysfunction, the injury of basal ganglia can also lead to impairments of visual formation, memory function and executive function that involve complex functional changes in brain regions (Bostan et al., 2010; Rodriguez-Sabate et al., 2017). Basal ganglia is well-connected to nearly all major cortical areas through cortico-basal ganglia-thalamus-cortical loops (Afifi, 2003). Therefore, significant interaction observed between the group and the frequency band in temporal and occipital areas in our study suggested that functional reorganization of these regions occurring after BGIS may lead to the above dysfunctions.

In addition, the post-hoc *t* test of the main effect of group showed that the DC values of the BGIS patients decreased in the right rolandic operculum in the slow-4 band and in the right superior temporal gyrus in the slow-5 band, compared with HCs. These results reflected that abnormal brain function activities of brain regions among BGIS patients in the acute stage. The role of the rolandic operculum is related to emotional processing and psychology (Gebauer et al., 2014; Marshall et al., 2019). Sutoko et al., (2020) used the k-means clustering method within one hundred sixty-five stroke patients and found that lesions in the right rolandic operculum contributed to worse psychological conditions (high apathy, depression, anxiety, and perceived stress). An fMRI study demonstrated that neuro feedback training in stroke patients with aphasia has resulted in increased activity in the rolandic operculum (Sreedharan et al., 2020). In addition, the right superior temporal gyrus is a critical region for auditory information processing (Yi et al., 2019) and is also associated with cognition function (Li et al., 2020c). A rs-fMRI study on 30 patients with post-stroke cognitive impairment showed that the cognitive function recovered significantly after repetitive transcranial magnetic stimulation (rTMS), and the neural activity of the superior temporal gyrus was higher than the control group (Li et al., 2020c). The abnormal brain function activities of the rolandic operculum and right superior temporal gyrus may induce cognitive and emotional impairments in BGIS patients.

### Correlation analysis

Correlation analysis showed that the decreased DC values in the right inferior temporal gyrus in slow-4 band were correlated with clinical indicators (uncorrected), but this correlation was no longer significant after Bonferroni correction. The inferior temporal gyrus is associated with visual object recognition (Hamamé et al., 2012), language comprehension (Bonilha et al., 2017) and emotion regulation (Deng et al., 2013). Neuroimaging revealed that the inferior temporal gyrus is connected to other cortical brain regions via white matter tracts, such as U-fiber and arcuate fasciculus (Lin et al., 2020). In addition, an fMRI study of 64 subcortical stroke patients found decreased static VMHC and increased dynamic VMHC variability in inferior temporal gyrus compared with HCs, but no significant correlations between static/dynamic VMHC and NIHSS, FMA were observed (Chen et al., 2021). Compared with HCs, abnormal dynamic characteristics of brain activity were detected in subacute stroke patients (Chen et al., 2018). A previous study found that patients with motor deficits exhibited significantly lower connectivity than controls within hours post-stroke, and recovered patients (NIHSS = 0) exhibited normal motor connectivity after 90 days (Golestani et al., 2013). Moreover, the visual network was activated in recovery from sensorimotor stroke (Archer et al., 2016). Based on these prior studies, we discreetly speculated that the decreased DC value in BGIS patients indicated that decreased brain functional activity of the right inferior temporal gyrus and reduced connections within other brain regions may aggravate the motor, language, and cognitive dysfunctions in patients. However, the neurological mechanism underlying the abnormal function of the right inferior temporal gyrus after BGIS needs further exploration. In addition, the NIHSS scores represent different neurological deficits (Kasner, 2006), and future research needs to study patients with different neurological dysfunctions separately.

### DC changes in acute BGIS patients are frequency specific

In the present study, the correlation between DC values and clinical indicators was only found in slow-4 band, suggesting that the DC changes of BGIS were frequency specific. Although the origins and functional significance of slow-4 and slow-5 bands remain unclear, studies suggested that slow-4 frequency oscillations are more sensitive to subcortical regions, especially in the basal ganglia (Yu et al., 2014; Zuo et al., 2010). Previous studies also suggested that the functional connections of brain had different frequency specific characteristics in neurological diseases. For example, Egorova and his colleagues observed that the fractional ALFF (fALFF) values of patients with post-stroke depression were correlated with the severity of depression in the slow-4 band, but did not correlate in the slow-5 band (Egorova et al., 2017). Moreover, a rs-fMRI study suggested that the use of specific frequency bands could be helpful in detecting neural changes in stroke patients with different outcomes in hand function (Zhao et al., 2018a). PD without depression patients exhibit widespread brain function activities in the slow-4 band (Liao et al., 2021) and the slow-4 band may be more sensitive in detecting functional connectivity alterations in patients with subclinical language deficit after stroke (Mohanty et al., 2019), indicating that slow-4 is more sensitive in detecting abnormal brain function activities in patients. Thus, our results implied that the slow-4 band can provide more in-depth clinical information for BGIS patients.

### Limitation

Our study has several limitations worth noting. First, the current findings may be limited by the relatively small sample size because of the strict inclusion and exclusion criteria. Second, this study is a cross-sectional study and did not follow dynamic brain functional changes in BGIS patients. In future studies, larger sample sizes and/or longitudinal data will be helpful to fully understand the neuroimaging mechanism of BGIS. Third, although patients with definite vascular stenosis or occlusion were excluded, the perfusion abnormalities can also occur in patients without vascular stenosis. Thus, the results should be treated with caution and further study should measure hemodynamic lags maps from resting-state fMRI data to correct these effects (Erdoğan et al., 2016; Tong et al., 2019). Furthermore, lesion flipping was conducted for statistical analysis, which might neglect the different brain functions/changes resulted from lesions in different hemispheres. Therefore, we shared the original DC maps without lesion flipping for future studies (http://www.restfmri.net/BGIS_BeforeFlipInThreeBands.zip).

## Conclusion

This resting-state fMRI study provided evidence for the functional abnormalities in local brain regions of the acute BGIS patients. A significant frequency band and group factor effect was observed in the bilateral cortical brain region, such as frontal lobe, temporal lobe, and occipital lobe. Moreover, the right inferior temporal gyrus may be the key hubs to evaluate stroke severity in patients with acute BGIS. The DC changes in acute BGIS patients were frequency specific, the slow-4 band can provide more in-depth clinical information for BGIS patients. Functional abnormalities in local brain regions may help us to understand the neurobiological mechanism of brain functional reorganization of BGIS patients and provide new ideas and insights for the targeted treatment for patients.

### Supplementary Information

Below is the link to the electronic supplementary material

ESM 1 (DOCX 549 KB)

## Data Availability

The data that support the findings of this study are available from the corresponding author, Zhijian Liang, upon reasonable request.
