# Supplementary material for: Frequency specific alterations of the degree centrality in patients with acute basal ganglia ischemic stroke: a resting-state fMRI study
Source: Brain Imaging Behav. 2023 Oct 12;18(1):19–33. doi: 10.1007/s11682-023-00806-1 (PMC10844151; doi:10.1007/s11682-023-00806-1)
Supplement: Supplementary file 1 — (DOCX 549 KB) [file 11682_2023_806_MOESM1_ESM.docx]

BIOR-D-22-00216

The supplementary materials:


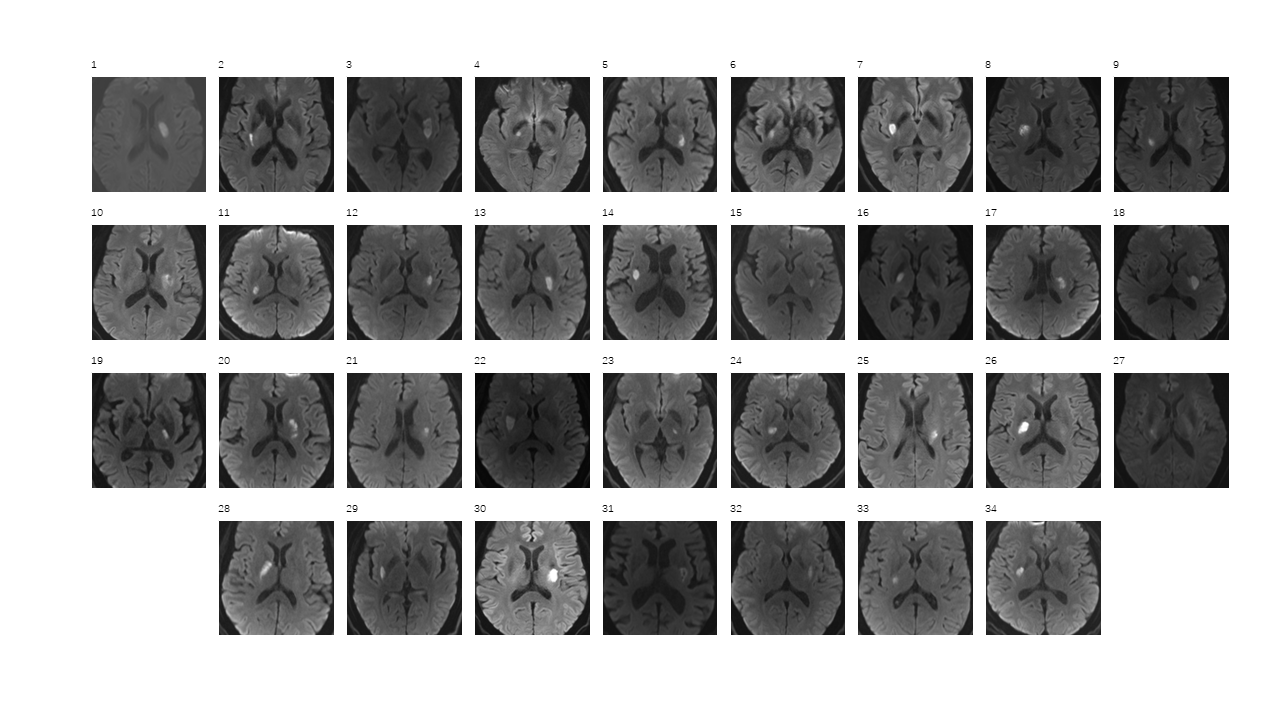


Figure S1. The map of stroke lesion of 34 BGIS patients.

BGIS, basal ganglia ischemic stroke.
